# Estimation of soil erodibility in Peninsular Malaysia: A case study using multiple linear regression and artificial neural networks

**DOI:** 10.1016/j.heliyon.2024.e28854

**Published:** 2024-03-29

**Authors:** Muhammad Ali Rehman, Norinah Abd Rahman, Ahmad Nazrul Hakimi Ibrahim, Norashikin Ahmad Kamal, Asmadi Ahmad

**Affiliations:** a Department of Civil Engineering, Faculty of Engineering and Built Environment, Universiti Kebangsaan Malaysia, 43600, UKM Bangi, Selangor, Malaysia; b Smart and Sustainable Township Research Centre, Universiti Kebangsaan Malaysia, 43600, UKM, Bangi, Selangor, Malaysia; c School of Civil Engineering, College of Engineering, Universiti Teknologi MARA, 40450, Shah Alam, Selangor, Malaysia; d Water Resource Management & Hydrology, Department of Irrigation and Drainage, 50480, Kuala Lumpur, Malaysia

**Keywords:** Erodibility, Computational methods, Predictive models, Artificial Neural Network (ANN), Multiple linear regression (MLR), Principal Component Analysis (PCA)

## Abstract

Soil erodibility (K) is an essential component in estimating soil loss indicating the soil's susceptibility to detach and transport. Data Computing and processing methods, such as artificial neural networks (ANNs) and multiple linear regression (MLR), have proven to be helpful in the development of predictive models for natural hazards. The present case study aims to assess the efficiency of MLR and ANN models to forecast soil erodibility in Peninsular Malaysia. A total of 103 samples were collected from various sites and K values were calculated using the Tew equation developed for Malaysian soil. From several extracted parameters, the outcomes of correlation and principal component analysis (PCA) revealed the influencing factors to be used in the development of ANN and MLR models. Based on the correlation and PCA results, two sets of influencing factors were employed to develop predictive models. Two MLR (MLR-1 and MLR-2) models and four neural networks (NN-1, NN-2, NN-3, and NN-4) optimized using Levenberg-Marquardt (LM) and scaled conjugate gradient (SCG) were developed and evaluated. The model performance validation was conducted using the coefficient of determination (R^2^), mean squared error (MSE), root mean squared error (RMSE), and Nash-Sutcliffe efficiency coefficient (NSE). The analysis showed that ANN models outperformed MLR models. The R^2^ values of 0.446 (MLR-1), 0.430 (MLR-2), 0.894 (NN-1), 0.855 (NN-2), 0.940 (NN-3), and 0.826 (NN-4); MSE values of 0.0000306 (MLR-1), 0.0000315 (MLR-2), 0.0000158 (NN-1), 0.0000261 (NN-2), 0.0000318 (NN-3), and 0.0000216 (NN-4) suggested the higher accuracy and lower modelling error of ANN models as compared with MLR. This study could provide an empirical basis and methodological support for K factor estimation in the region.

## 1 Introduction

Soil erosion is an indigenous geological phenomenon of separation and transportation of soil particles from their origin, and their accumulation either onsite or offsite [1,2]. Soil erosion induced by excessive precipitation and augmented surface runoff together with anthropogenic inference such as urbanization, deforestation, and agriculture lead to land degradation, reduced quality and quantity of arable land, enhanced river sedimentation, and prompting stern geological hazards [[3], [4], [5]]. Although, the erosion problem occurs along the globe, however, countries with tropical and subtropical climates, such as Malaysia, are particularly vulnerable to soil erosion and its associated challenges due to high rainfall intensities in these regions [6,7].

Adopting suitable soil management practices is the best possible way to prevent undesirable impacts of soil erosion on the environment. The prospective risk of soil erosion and its spatial distribution are required to be estimated for effective conservation of soil [8,9]. Various empirical models for soil erosion assessment have been established, among which the Universal Soil Loss Equation (USLE) and Revised Universal Soil Loss Equation (RUSLE) are broadly implemented around the world [10]. The estimation of soil erosion through these models requires the determination of a few factors including rainfall erosivity, soil erodibility, terrain, land use management, and conservation practice [11].

Soil erodibility, typically termed as the K-factor, is an inherent property of soil that determines its resistance to both detachment and transport [2]. K factor is a crucial marker of soil erosion susceptibility [12] and is a vital parameter employed in various soil erosion estimation models influencing the extent of soil loss [13,14]. The concept of the K factor introduced in the USLE and RUSLE models defines it as the quantity of soil loss due to rain splash, runoff, or infiltration within a standard unit plot [15,16]. Although the field measurement by establishing the standard unit plots yields a more precise estimation of soil erodibility, however, longer evaluation periods are required, thus proving it to be time-consuming, laborious, and costly with fractional spatial illustration [17]. Therefore, various studies have been focused on developing empirical models to estimate soil erodibility based on physical and chemical properties, including mathematical, graphical, and instrumental methods [18].

Various techniques and methods have been employed in evaluating the effect of soil properties on the K factor. With the advancement in computing tools, researchers have turned their attention to involving numerical, statistical, and machine learning algorithms in predictive modelling. Due to their ability to address challenging problems, these methods have the potential to develop regional or local soil erodibility indices, by developing a relationship between the K factor and conveniently attainable soil parameters in order to forecast soil erodibility [19]. Predictive modelling approaches such as multiple linear regression (MLR), artificial neural networks (ANNs), adaptive neuro-fuzzy inference systems (ANFIS), support vector machines (SVM), random forest (RF) technique, and regression trees are some of the technologies which have been commonly employed for developing models for estimating soil properties [[20], [21], [22], [23], [24], [25]].

In comparison with other advanced machine learning techniques, MLR and ANN have been most widely used in prediction modelling due to their adaptability, effectiveness, and usability. These traits make them one of the most reliable techniques for predicting measurable and continuous variables [26]. MLR and ANNs have been found helpful in estimation and forecasting various parameters in the fields of civil engineering, geotechnical engineering, water engineering, hydrology, and environmental geology [[27], [28], [29], [30], [31]].

The nature of ANN models is analogous to the human brain and is devised based on the principle of simulating several interconnected neurons which consist of input layers, output layers, and hidden layers [24]. ANNs have acquired extensive recognition for their capability to model intricate and non-linear relationships [32] and have helped foretell practical and cost-effective ways to solve problems [33]. On the other hand, MLR proved to have advantages such as simplified calculations, efficient descriptive power, and convenience in usability [32]. These computational methods (MLR and ANNs) for forecasting and developing estimation models have been found useful in the prediction and assessment of soil erosion and erodibility [20,21,[34], [35], [36], [37], [38], [39], [40], [41], [42], [43], [44]].

Peninsular Malaysia has a tropical monsoon climate with a high average annual precipitation, thus making this area vulnerable to soil erosion. Even though, various studies have been carried out on the assessment and mapping of soil erodibility, a noticeable void prevails in the literature on thorough studies using sophisticated computational techniques for the estimation of soil erodibility in Peninsular Malaysia. This highlights the necessity for research endeavours to employ computational methods, such as MLR and ANN, for developing soil erodibility estimation models based on diverse influencing factors for better understanding and assessment of erodibility in Peninsular Malaysia.

To address the aforementioned necessity, the present case study reports the estimation of soil erodibility, which demonstrates the proneness to soil erosion, by employing a sophisticated computational approach. For this purpose, laboratory experimental analysis was carried out on soil samples obtained from various locations in Peninsular Malaysia. Further, by utilizing these experimental results and additional indices for quantifying erodibility indicated in the literature [6,32,41,[45], [46], [47]], a detailed statistical analysis was carried out to identify the most influencing parameters from the selected dataset, erodibility estimation models were developed. The K factor was simulated using MLR and ANN with the objective of assessing the efficiency and reliability of predictive models for the estimation of soil erodibility based on selected parameters concerning the local or regional conditions. Findings from this study would be significant in providing necessary help in selecting appropriate soil parameters and estimation models for the assessment of erosion risk, mitigation, and hazard management planning.

## 2 Materials and methods

### 2.1 Soil sampling and data acquisition

**Fig. 1:**
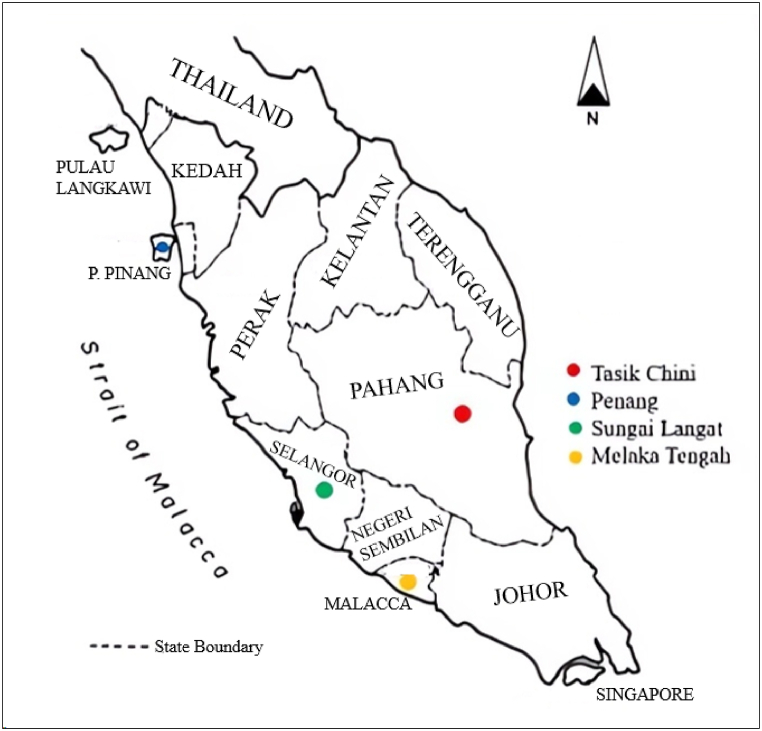
Location of the study sites in Peninsular Malaysia.

Peninsular Malaysia, a Southeast Asian region, lies in the tropical latitude having substantial precipitation, high humidity, and temperature dominated by southwest (May to September) and northeast (November to March) monsoons. Since Peninsular Malaysia is located near the equator, its climate is generally categorized as equatorial having uniformly high temperature with an average of about 27 °C [48]. The Peninsular Malaysia receives a mean annual rainfall of approximately 2540 mm [49]. The soil samples were collected from different sites, based on the available soil series information [9,[50], [51], [52]] situated in Peninsular Malaysia, including Tasik Chini (3°26′00” N - 102°55′00” E) in Pahang state, Penang (Pulau Pinang) Island (5°22′02.3” N - 100°14′55” E) in Penang state, Melaka Tengah District (2°15′00” N - 102°15′00” E) in Melaka state, and across Sungai Langat (2°55′56.9” N - 101°46′34.9” E) in Selangor state near Universiti Kebangsaan Malaysia. The study sites are shown in Fig. 1.

Soil sampling was carried out at the study sites and the disturbed soil samples were collected with the help of auger. The samples were immediately sealed in plastic bags and were carefully transported to the laboratory for subsequent experimental analysis. A total of 103 soil samples were extracted from the abovementioned study sites. In this study, 14 influencing factors, including gravel coverage (G), very coarse sand (VCS), coarse sand (CS), medium sand (MS), fine sand (FS), very fine sand (VFS), silt (Si), clay (C), volumetric water content (w), organic matter (OM), critical level of organic matter (CLOM), modified clay ratio (MCR), ROM erodibility index (EI_ROM_), and critical shear stress (***τ***_c_) were chosen for estimation modelling of the K factor. The majority of the chosen parameters are related to soil particle distribution, which if known, can be directly calculated by soil particle distribution outcomes.

### 2.2 Determination of the K factor and influencing factors

Researchers have endeavoured to devise simple indices and empirical models to estimate soil erodibility. Amongst these thorough empirical studies, the Tew equation [53] has been identified to yield appropriate estimates of the soil erodibility for Malaysian soil series. Consequently, the Tew equation is employed in this study in line with the recommendations of the Department of Irrigation and Drainage (DID), Malaysia [54]. The equation is expressed as:

(1) $$K=\left[1.0\times 10^{-4}\left(12-OM\right)M^{1.14}+4.5\left(s-3\right)+8.0\left(p-2\right)\right]/100$$

where K represents soil erodibility in [*(ton/ac.) (*100 ft*.ton.in/ac.hr)*], OM represents soil organic matter content, and M in the equation can be described as a particle size parameter which can be determined as [(% silt + % very fine sand) (100 - % clay)], s represents the soil structure code, and p represents the soil permeability code. The K factor can be multiplied with a conversion factor of 1/7.59 to convert the units of the K factor to SI units [*(ton/ha) (ha.hr/MJ.mm)*].

**Fig. 2:**
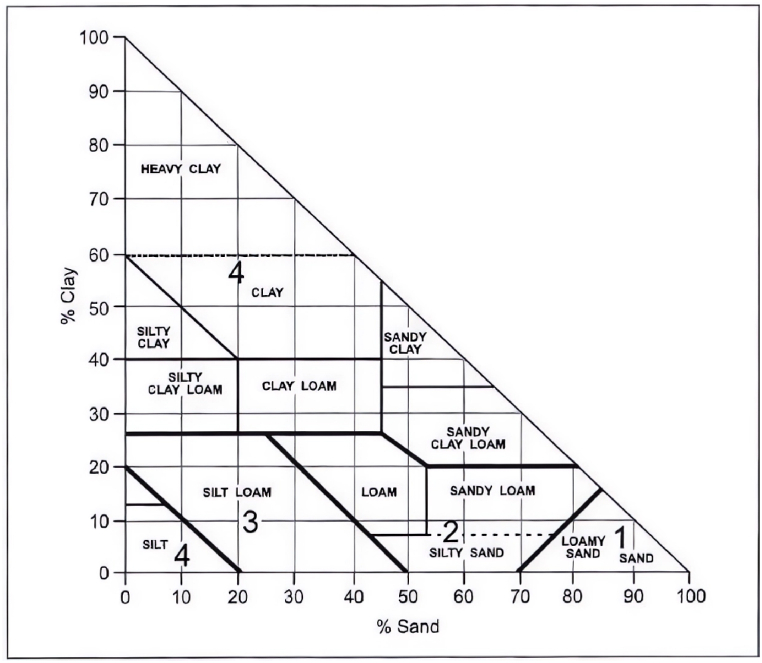
Soil Structure Code based on Textural Triangle [54].

Laboratory experiments, encompassing sieve analysis, hydrometer analysis, and organic matter content test were performed to find out the parameters used in equation (1). The grain size distribution analysis of the soil was carried out in accordance with BS 1377-Part 2:1990. The soil samples were oven-dried and were thoroughly crushed to break down any lumps. Dry sieving was carried out to determine the distribution of coarse particles. Soil samples were kept in a stack of sieves and the sieves were shaken with the help of a mechanical sieve shaker at a constant rate. The weight of retained soil at each sieve was measured and recorded. Hydrometer analysis was carried out to determine the distribution of fine particles. The soil passing through a 63-μm sieve was separated and 50 g of the representative sample was taken for the hydrometer analysis. For every soil sample, the grain size distribution curves were developed, and the percentages of clay, silt, and sand-sized fractions were determined. The organic matter (OM) content of the soil was determined by treating the soil with a 30% solution of hydrogen peroxide (H_2_O_2_). For this test, 10 g of the representative soil passing through a 2 mm sieve was taken in a beaker. Then, 30 ml of 30% hydrogen peroxide solution was poured into the beaker and warmed gradually up to 60 °C. Heating was carried out for about 3 h until the moment when pouring another 10 ml of 30% hydrogen peroxide solution did not develop any gas bubbles. After that, the soil solution was boiled to obtain a slurry of dense consistency. Then the beaker was placed in the oven to dry the solution to a constant mass at 105 °C for 16 h. The mass before pouring of solution and after drying were recorded and OM content was calculated. United States Department of Agriculture (USDA) classified soil into twelve textural classes, each having distinctive characteristics [55]. Soil structure code can be determined through the textural triangle, as shown in Fig. 2 [54]. After determining the structure code, the soil permeability code can be correlated with the structure code in accordance with Table 1 [2]. Further, the values of the abovementioned all parameters were then put in equation (1) to calculate the K factor value.

**Table 1:** Soil Permeability Code [2].

Soil Texture	Permeability Code
Sand	1
Sandy Loam, Loamy Sand	2
Loam, Silt Loam	3
Clay Loam, Sandy Clay Loam	4
Silty Clay Loam, Sandy Clay	5
Clay, Heavy Clay	6

The parameters related to soil particle size and texture, including G (>2.00 mm), VCS (2.00–1.00 mm), CS (1.00–0.5 mm), MS (0.5–0.25 mm), FS (0.25–0.1 mm), VFS (0.1–0.05 mm), Si (0.05–0.002 mm), and C (<0.002 mm), were determined from particle size distribution based on USDA classification [55]. CLOM also serves as an indicator of soil's vulnerability to erosion reflecting the capability of aggregate formation of soil which resists the detachability [46]. It can be determined by the formula shown in Equation (2). MCR is another index of soil erodibility, referring to the binding of soil particles to resist detachment [45]. It can be determined by the formula shown in Equation (3).

(2) CLOM=OM/(clay+silt)

(3) MCR=(%sand+%silt)/(%clay+%OM)

EI_ROM_ is an index, developed specifically for the Malaysian soil series by the researchers Roslan Zainal Abidin and Mazidah Mukri, therefore named as ROM scale [56], that demonstrates the level of erosion by considering the soil textural composition [6]. It can be determined by the formula shown in Equation (4). ***τ***_c_ is an important parameter of soil in governing the soil erosion process. The particles begin to move and detach under the influence of erosion agents if the critical shear stress of the soil exceeds [57]. It can be determined by the formula as shown in Equation (5).

(4) $${EI}_{ROM}=\left(\%sand+\%silt\right)/\left(2\times \%clay\right)$$

(5) $$\tau_{c}=0.1+0.1779\left(SC\right)+0.0028{\left(SC\right)}^{2}-2.34\times 10^{-5}{\left(SC\right)}^{3}$$

where SC in equation (5) is the combined percentage of silt and clay in decimal fractions.

### 2.3 Data statistics and validation

The descriptive statistics of the variables explaining mean, median, minimum, maximum, standard deviation, standard error, and sample variance were determined to demonstrate the spatial variability and distribution of data. The significance of relationships between the K factor and the variables was found using Pearson correlation analysis. Further, the potential variables significantly influencing the K factor were identified by conducting principal component analysis (PCA). About two-thirds of the sampled data points were utilized for developing the model and training, while the remaining one-third were used for validation and testing of the developed models.

The developed estimation models are often validated and tested for reliability and performance since the predicted values by the estimation models may not always be consistent with the original calculated values. These performance and reliability checks help in the selection of appropriate predictive models. In this study, the validation of the estimation models was performed utilizing metrics such as coefficient of determination, also called R-squared value (R^2^), mean squared error (MSE), root mean square error (RMSE), and Nash-Sutcliffe efficiency coefficient (NSE). The equations for MSE (equation (6)), RMSE (equation (7)), and NSE (equation (8)) are as follows:

(6) $$MSE=\frac{1}{n}\sum_{i=1}^{n}{\left(K_{est,i}-K_{cal,i}\right)}^{2}$$

(7) $$RMSE=\sqrt{\frac{1}{n}\sum_{i=1}^{n}{\left(K_{est,i}-K_{cal,i}\right)}^{2}\;}$$

(8) $$NSE=1-\;\frac{\sum_{i=1}^{n}{\left(K_{est,i}-K_{cal,i}\right)}^{2}}{\sum_{i=1}^{n}{\left(K_{cal,i}-\bar{K_{cal}}\right)}^{2}}$$

where n is the number of data observations, $K_{cal,i}$ is the calculated K factor from original data at *i* location, $K_{est,i}$ is the estimated K factor at *i* location, and $K_{cal}$ is the calculated mean K factor.

### 2.4 MLR and ANN modelling

MLR modelling is one of the most widely used soft computing concepts in a variety of research areas. Since it is straightforward to employ, it does not require specialized skills and high computational power, thus people lacking thorough knowledge of artificial intelligence can easily implement MLR without needing sophisticated computing systems. Its simplicity and flexibility at the same time allow several input parameters to concurrently contribute to predictive modelling. MLR models can be represented mathematically as:

(9) $$y=\beta_{0}+\sum_{i=1}^{n}\beta_{i}.x_{i}$$

where y represents the output or dependent variable, $\beta_{0}$ is the intercept, $\beta_{i}\to \beta_{n}$ are the regression coefficients of input variables, and $x_{i}\to x_{n}$ denote the input variables [58].

**Fig. 3:**
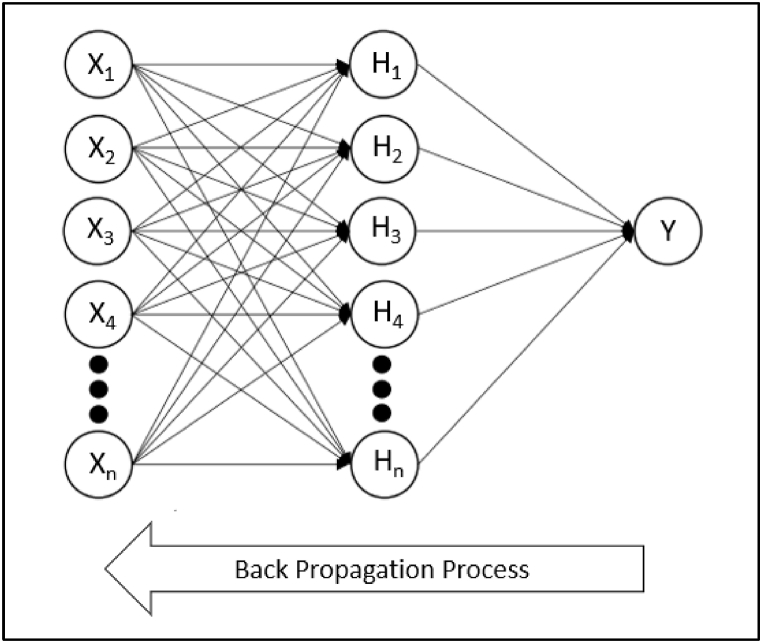
Basic ANN architectural model.

**Fig. 4:**
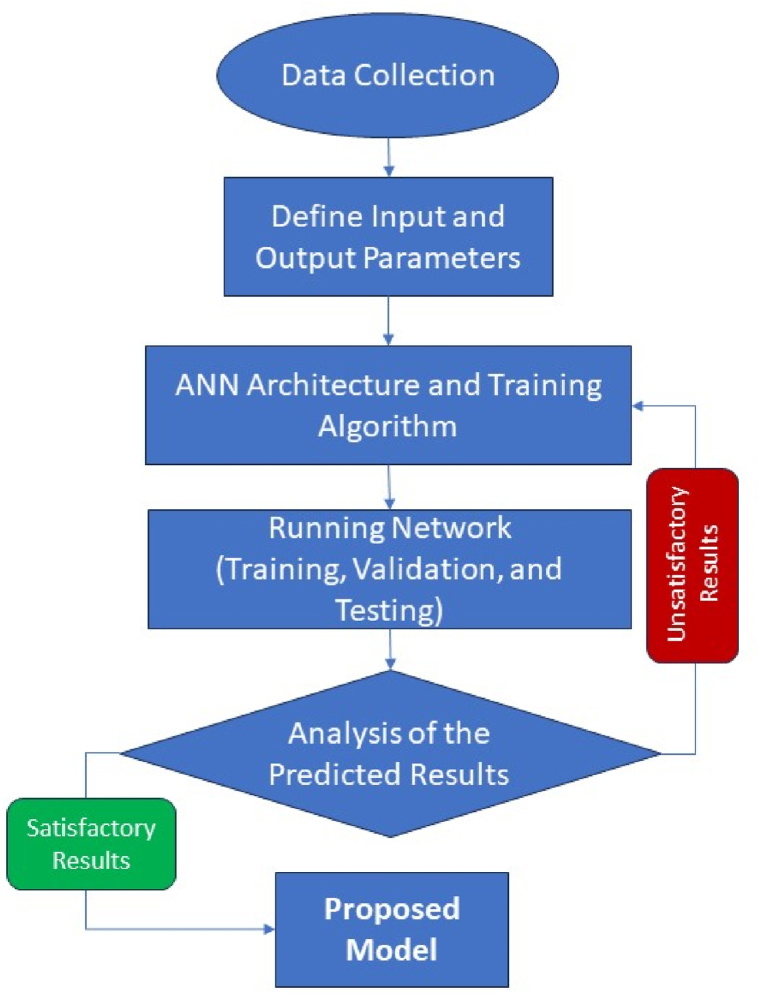
ANN model development flowchart.

**Fig. 5:**
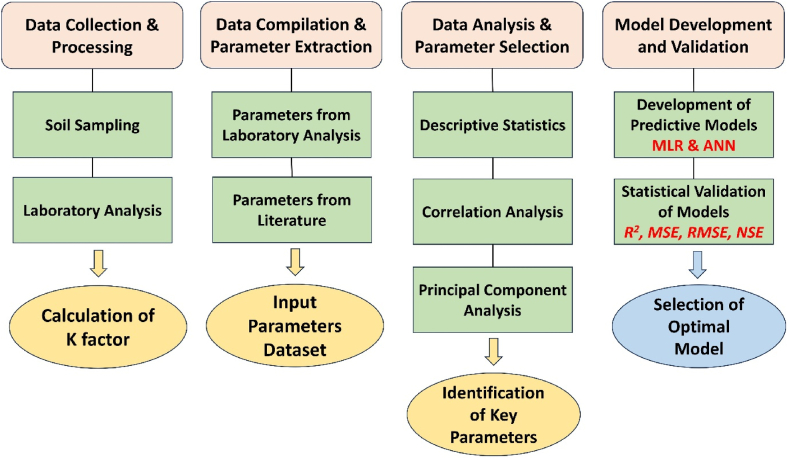
Graphical Representation of the Methodology Employed in this Study.

In the era of technological evolution, estimation modelling of earth processes and natural hazards using intelligent-based systems like ANN is seen as crucial. ANNs have found multiple uses across a variety of research domains as they assist in solving complex and varied dataset problems [59]. ANNs are considered capable to learn, comprehend, and convey significant insight regarding a furnished dataset [26] since they emulate the human brain's biological structure by having a network of sensitive interconnected neurons [41]. The neural network consists of a series of layers where parallel systems are made up of numerous neurons that are interconnected to one another to form elementary processors. Generally, a neural network contains three layers: input layer, hidden layers, and output layer, as shown in Fig. 3. The network operates through the connection of these elements in such a manner that each neuron relates to all the neurons from the previous layer to develop a model that ensures the desired output by involving inputs, their weights, and transfer functions [60]. The development model of the feedforward-backpropagated multilayer perceptron neural network is shown in Fig. 4. Since ANNs have the flexibility and ability to handle linear and nonlinear variable interactions, that makes them an appropriate choice for the predictive modelling of a substantial number of variables. The graphical representation of the methodology used in this study is illustrated in Fig. 5.

In this study, the primary data processing was performed using Microsoft Excel-365. Subsequent statistical analyses, including Pearson correlation analysis, PCA, and MLR were conducted using IBM-SPSS 27. The ANN model development and analysis were carried out using MATLAB R2017b. Two training algorithms, Levenberg-Marquardt (LM) and Scaled Conjugate Gradient (SCG), were employed in ANN model development and analysis. Only the iterations with optimum performance were selected.

## 3 Results and discussions

### 3.1 Basic characteristics of variables

The descriptive statistics of the K factor and other influencing variables is represented in Table 2. The minimum and maximum values of K in this study were 0.004 and 0.039 t ha h.ha^−1^.MJ^−1^.mm^−1^, respectively, with a mean value of 0.023 t ha h.ha^−1^.MJ^−1^.mm^−1^. The median K value was closer to the mean value, thus representing the normal distribution of the K factor. The Coefficient of Variation (CV) is a crucial measure in discerning the distribution and spatial variability of the variables in a dataset. The spatial variability of the data can be categorized as weak, moderate, or strong if the CV is less than 10%, between 10 and 100%, and greater than 100%, respectively [61]. The K factor in the study exhibited a small standard deviation (SD), and the CV ranged between 10% and 100%, suggesting a weak to moderate K factor spatial variability. The distortion or asymmetry of the data deviating from normal distribution can be measured by skewness [62]. A dataset may be considered as normally distributed if the skewness value ranges from −1 to 1 [11,63]. The negative and positive values of skewness represent that data is left-skewed and right-skewed, respectively. If the values are lower than −1 and higher than 1, the data is considered to be highly skewed. The value of skewness for the K factor in this study was −0.262, thus representing the data to be almost symmetrical. The presence of a total degree of outliers in the data can be represented by kurtosis [62]. For a symmetric distribution of data, a value of 3 for kurtosis is generally expected [61]. A value greater than 3 shows positive kurtosis whereas a value lower than 3 shows negative kurtosis, representing light-tailed top peaked distribution and heavy-tailed flatter distribution, respectively [2]. The value of kurtosis for the K factor in this study was −0.075, thus indicating negative kurtosis.

**Table 2:** Summary of descriptive statistics.

	Minimum	Maximum	Mean	Median	SD	CV	Kurtosis	Skewness
G	1.014	45.938	25.858	28.093	13.089	46.591	−1.065	−0.368
VCS	3.279	25.437	16.451	17.192	4.912	28.570	−0.468	−0.366
CS	5.861	29.497	15.381	15.424	4.023	26.083	0.775	0.395
MS	7.871	40.018	16.810	15.222	6.316	41.494	1.573	1.103
FS	3.950	46.247	17.092	15.463	8.592	55.563	0.615	0.807
VFS	0.496	20.063	4.297	3.172	3.942	124.297	4.522	2.043
Si	0.120	10.378	2.455	1.864	1.861	99.818	6.766	2.369
C	0.047	9.404	1.658	1.320	1.443	109.252	13.269	3.119
w	6.924	71.163	22.580	19.741	11.413	57.814	5.231	2.049
OM	0.023	5.284	1.085	0.745	1.080	145.019	4.615	2.098
CLOM	0.015	2.306	0.319	0.210	0.345	164.114	14.062	3.167
MCR	4.501	246.419	40.013	32.725	31.819	97.231	18.041	3.521
EI_ROM_	3.488	641.114	43.305	25.816	75.938	294.145	44.903	6.369
τ_c_	0.102	0.131	0.107	0.106	0.005	4.789	6.503	2.244
K	0.004	0.039	0.023	0.022	0.007	33.698	−0.075	−0.262

**Fig. 6:**
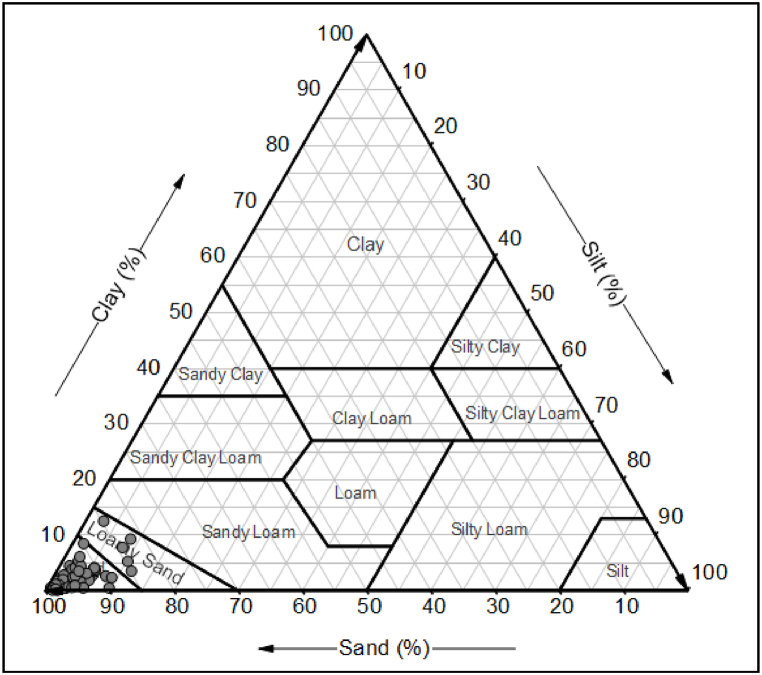
Ternary Plot of the Texture Distribution of Soil Samples based on USDA Criteria.

The mean values of clay and silt content in the soil were 1.66% and 2.45%, ranging from 0.05 to 9.40% and 0.12–10.4%, respectively. The mean OM value was 1.085%, ranging from 0.023 to 5.28%. The collected soil samples were dominated by sand fraction; thus, the majority of the soils were classified as sands or loamy sands according to USDA textural classification. The soil textural classification is represented in Fig. 6, showing the concentration of soil samples in the sand textural class.

### 3.2 Determination of potential variables

**Fig. 7:**
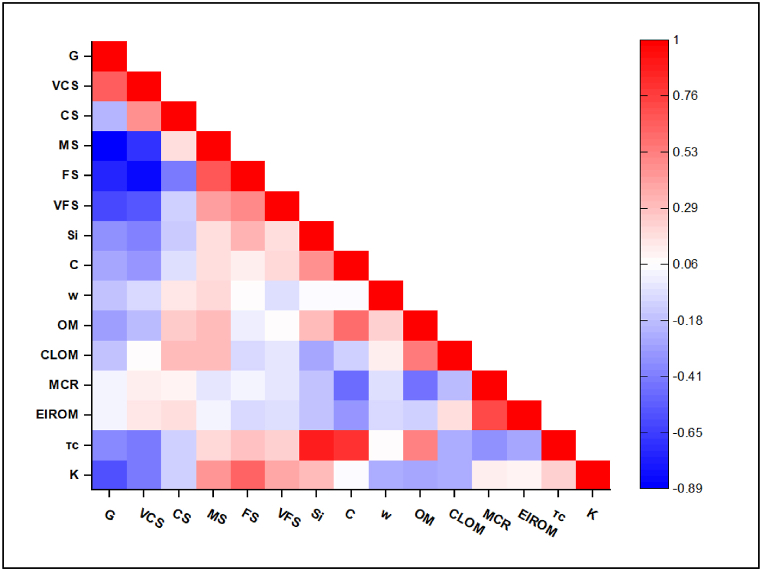
Pearson correlation matrices.

The intrinsic properties of soil and the extrinsic conditions notably affect the soil erodibility. In this study, 14 variables related to soil physical and chemical properties were extracted. Pearson correlation analysis was carried out to determine the relationship between the K factor and extracted variables. The Pearson correlation matrix is shown in Fig. 7. Further, PCA was carried out to determine the principal components (PCs) of the main potential influencers for the estimation of the K factor.

Soil textural composition is an important characteristic in governing the soil infiltration and water retention properties and significantly affecting the capacity of soil to hold essential nutrients [64]. It can be observed from Fig. 6 that the K factor exhibited a significant positive correlation with MS, FS, VFS, and Si. This indicates that an increase in the percentages of MS, FS, VFS, and Si in the soil will make the soil more prone to erodibility, as reported by Huang et al. (2022), Shirzadi et al. (2022), and Alqadhi et al. (2023) [14,40,47]. Whereas the K factor was found to have a significantly negative correlation with G and VCS, indicating that high percentages of G and VCS in the soil would lower the rate of erodibility. This complies with the fact that as the size of the particles either decreases or exceeds the range of 20 μm–200 μm, the detachment of soil particles from their source decreases [[65], [66], [67]]. Soil particles smaller than 20 μm (or 0.002 mm) tend to develop cohesive forces that counter the particle separation, while particles larger than 200 μm (or 0.2 mm) are hard to detach and transport under the influence of erosion drivers due to their mass [11]. Therefore, the soils having particles in the size range of Si, VFS, and FS are prone to detachability. The K factor was found to be negatively correlated with OM, indicating that an increase in OM percentage in the soil will tend to lower the K factor values, as OM is considered to bind the soil particles together to resist detachability. Fine textured soils tend to have higher percentages of OM than sandy or loamy soils, as indicated in the previous studies by Gupta et al. (2010), Mallick et al. (2016), and Gyamfi et al. (2016) [[68], [69], [70]]. The same interrelation pattern between OM and C was observed in this study.

**Fig. 8:**
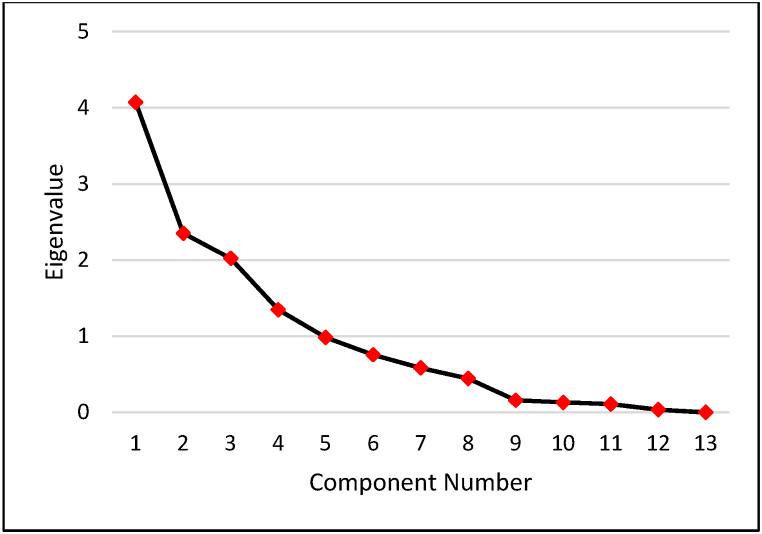
Scree Plot representing Components with Eigenvalues.

Further, to analyze the dimension reduction on the potential variables, PCA was carried out. The extraction of PCs was carried out based on the eigenvalues greater than 1. Fig. 8 shows the scree plot indicating variables against their eigenvalues. It can be observed that 4 PCs were extracted having eigenvalues greater than 1.

Table 3 represents the factor loadings of 4 PCs in the component matrix, all possessing eigenvalues above 1. It was observed that the percent cumulative contribution to variance was above 75% for the 4 PCs. From the component matrix, two sets of potential variables for estimation modelling of the K factor were selected. The first set of variables (Set-1) consisted of variables having the highest factor loadings in each PC, while the second set of variables (Set-2) included those with factor loading within the range of 10% of variation from the highest factor loading for each PC. For SET-2 variables, multicollinearity was also examined. The value of variance inflation factor (VIF) greater than 1 indicates a degree of multicollinearity [32]. It was observed that EI_ROM_ and MCR with R-value equal to 0.735 and VIF equal to 2.17, while VCS and FS with R-value equal to −0.857 and VIF equal to 3.76 are multicollinear. Thus, variables with higher factor loading were retained for estimation models. For PC3, only CLOM was eligible. Consequently, the selected predictive variables for K factor estimation modelling were Tc, FS, CLOM, and EI_ROM_ for Set-1, and ***τ***_c_, VCS, OM, CLOM, and EI_ROM_ for Set-2.

**Table 3:** Principal component analysis of influencing factors.

Influencing Factors	PC1	PC2	PC3	PC4
VCS	−0.789	0.519	−0.128	0.140
CS	−0.259	0.446	0.525	0.334
MS	0.570	−0.314	0.644	−0.029
FS	0.646	−0.651	0.142	−0.142
VFS	0.506	−0.401	0.152	−0.064
Si	0.698	0.076	−0.279	0.480
C	0.727	0.426	−0.168	0.174
W	0.148	0.155	0.326	−0.029
OM	0.531	0.600	0.443	0.097
CLOM	−0.051	0.298	0.800	−0.211
MCR	−0.468	−0.584	0.121	0.573
EI_ROM_	−0.400	−0.359	0.369	0.621
*τ*_c_	0.827	0.267	−0.269	0.404
Eigenvalue	4.073	2.353	2.022	1.348
Contribution to Variance (%)	31.328	18.102	15.557	10.368
Cumulative Contribution to Variance (%)	31.328	49.429	64.987	75.354

### 3.3 Estimation modelling

In this study, two sets of influencing variables were used to establish MLR and ANN estimation models for the prediction of the K factor. The parameters from set-1 and set-2 were used as predictive variables or independent variables, and the K factor was used as the simulation variable or target variable. Set-1 consisted of Tc, FS, CLOM, and EI_ROM_ as input variables, and the developed MLR model was designated as MLR-1. Whereas set-2 consisted of ***τ***_c_, VCS, OM, CLOM, and EI_ROM_ as input variables, and the developed MLR model was designated as MLR-2.

The developed MLR-1 and MLR-2 models are mathematically represented as equation (10) and equation (11), respectively:

(10) $$K=0.0050078+0.0005181\times \text{FS}-0.0044751\times \text{CLOM}+0.0000192\times {EI}_{ROM}+0.0860495\times \tau_{c}$$

(11) $$K=-0.0543488+0.8316173\times \tau_{c}-0.0005742\times VCS-0.0053456\times OM+0.0068464\times CLOM+0.0000154\times {EI}_{ROM}$$

**Fig. 9:**
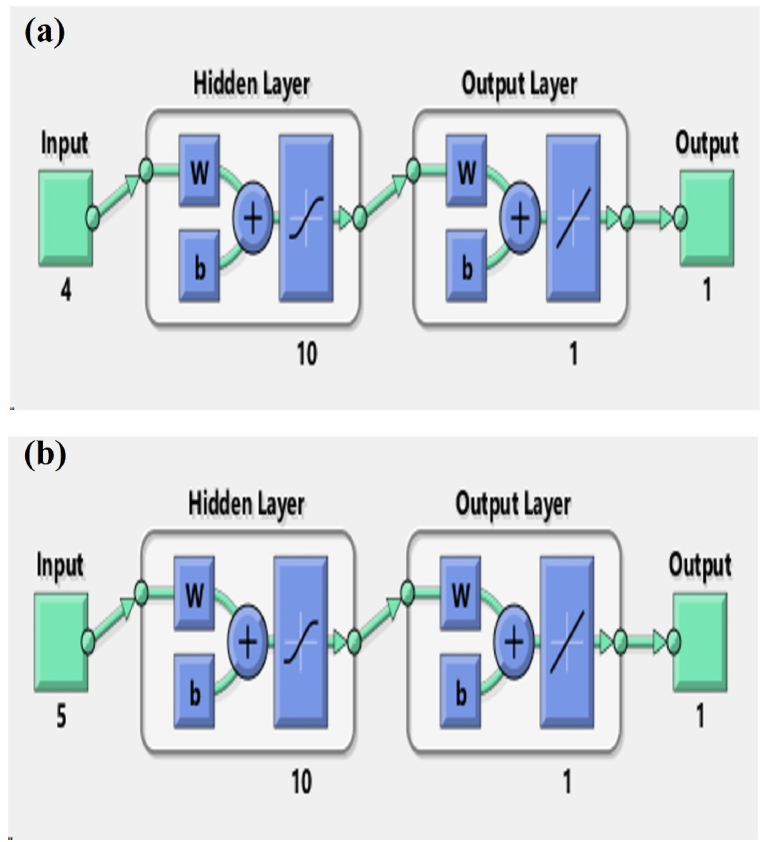
Layout of ANN models: (a) NN-1 & NN-2, (b) NN-3 & NN-4.

Four distinct feed-forward backpropagation neural network models (demoted as NN-1, NN-2, NN-3, and NN-4) were developed using neural net fitting tool (nftool) in the MATLAB to explore different possible options to visualize the efficacy and suitability of ANNs in predicting the K factor. The developed ANN models contained common attributes in terms of hidden layer architecture and transfer functions, while these models exhibited differences in terms of input parameter sets and training algorithm. The models NN-1 and NN-2 were configured with LM backpropagated training algorithm (TRAINLM) using input parameter set-1 and set-2, respectively. Whereas the models NN-3 and NN-4 were configured with SCG backpropagated training algorithm (TRAINSCG) using input parameter set-1 and set-2, respectively. The layout of models NN-1 and NN-3 is shown in Fig. 9(a), whereas Fig. 9(b) illustrates the layout of model NN-2 and NN-4. All the developed models comprised of only one hidden layer with 10 neurons to avoid overfitting the model. For all the neural network models in this study, nftool employed hyperbolic tangent sigmoid transfer function (TANSIG) to the hidden layer neurons, while linear transfer function (PURELIN) to the output layer. Similarly, LEARDGM (Gradient Descent with Momentum) and MSE (Mean Squared Error) were employed as the adaption learning and performance functions by the nftool. The summary of the description of ANN models is presented in Table 4.

**Table 4:** Description of ANN models.

Model	Input Variables	Partitioning Ratio *(Training-Validation-Testing)*	Training Algorithm	Hidden Layer Neurons	Transfer Function	Learning Adaption Function	Performance Function
NN-1	***τ***_c_, FS, CLOM, and EI_ROM_	70-10-20	TRAINLM	10	HL: TANSIG OL: PURELIN	LEARNGDM	MSE
NN-2	***τ***_c_, VCS, OM, CLOM, and EI_ROM_	70-10-20	TRAINLM	10	HL: TANSIG OL: PURELIN	LEARNGDM	MSE
NN-3	***τ***_c_, FS, CLOM, and EI_ROM_	70-10-20	TRAINSCG	10	HL: TANSIG OL: PURELIN	LEARNGDM	MSE
NN-4	***τ***_c_, VCS, OM, CLOM, and EI_ROM_	70-10-20	TRAINSCG	10	HL: TANSIG OL: PURELIN	LEARNGDM	MSE

Note: HL: Hidden Layer, OL: Output Layer.

**Fig. 10:**
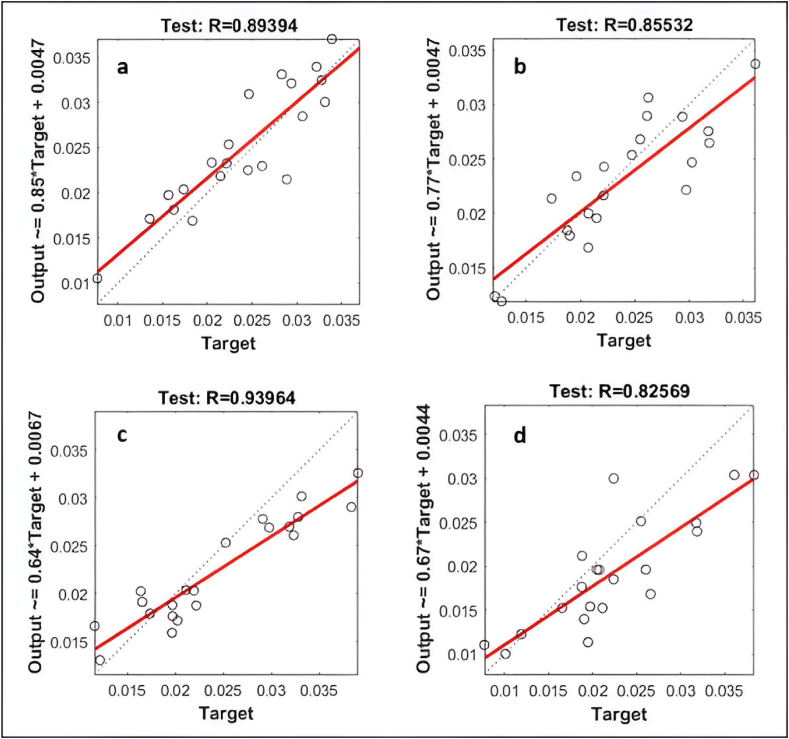
ANN model regression plots (a) NN-1, (b) NN-2, (c) NN-3, (d) NN-4.

**Fig. 11:**
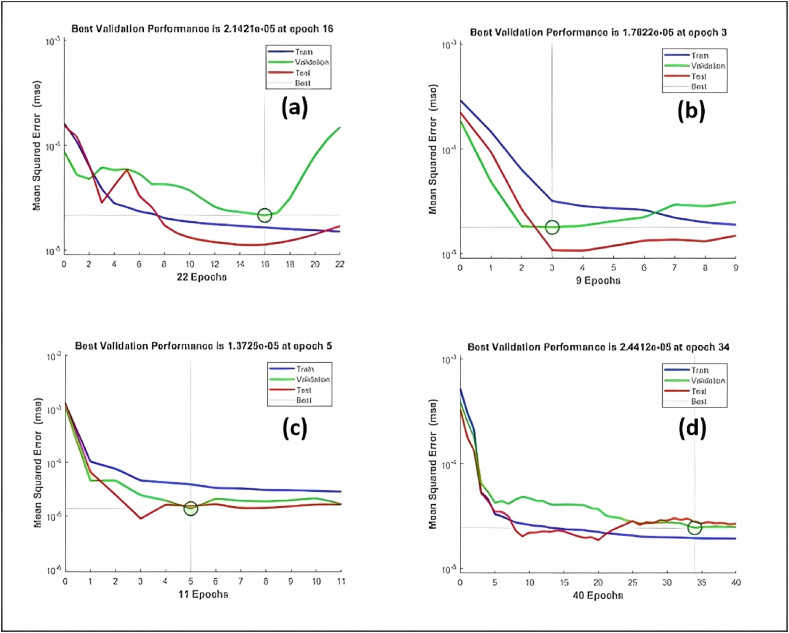
Ann model performance plots (a) NN-1, (b) NN-2, (c) NN-3, (d) NN-4.

The regression and performance plots of ANN models are presented in Fig. 10(a–d) and Fig. 11(a–d), respectively. The regression plots of the test sample data indicate the strength of the relationship between the target and the output. The R-squared values for NN-1, NN-2, NN-3, and NN-4 were observed to be 0.894, 0.855, 0.940, and 0.826, respectively. The value of the performance function in relation to the iteration number is displayed in the performance plot. Each repetition of the complete training set is referred to as an epoch. The network adjusts the weights automatically every epoch in a manner that reduces the error, therefore various epochs are often required before the completion of training. The generalization comes to an end as a rise in the mean square error (MSE) of the validation samples is observed, so at this moment the training automatically stops. Based on the training phase of ANN models, the importance of generalization and early stopping to avoid overfitting is highlighted. In terms of MSE values and corresponding epochs, the number of training epochs for each model and the best validation performance are achieved. The NN-1 model performed 22 epochs during the data processing and demonstrated the best validation performance in the 16th epoch with the lowest mean square error of 0.000021421. The best validation performance for the NN-2 model was observed in the 3rd epoch with the lowest MSE value of 0.00001782. Similarly, the best validation performance for NN-3 and NN-4 were observed in the 5th and 34th epoch, respectively, with the lowest MSE of 0.000013725 and 0.000024412.

The outcomes of the developed estimation models are in close agreement with the results presented in earlier studies. Yusof et al. (2014) [37] applied different ANN schemes to predict soil erodibility for the soil series data in Peninsular Malaysia, suggesting a good performance of the predictive model with an R^2^ value of 0.86. Ostovari et al. (2016) [19] aimed to develop pedotransfer functions (PTFs) to predict soil erodibility by using MLR, MFIS (Mamdani Fuzzy Inference System), and ANN. They concluded the ANN model to be the most optimal in predicting soil erodibility with an R^2^ value of 0.85, outperforming MLR and MFIS. Kouchami-Sardoo et al. (2020) [39] reported the performance of a multi-layer perceptron neural network in predicting soil erodibility. Their developed model performance, having an R^2^ value of 0.87, indicated a strong basis for erodibility prediction. Similarly, the studies conducted by Zhu et al. (2022) [32] and Pacci et al. (2023) [41] used the ANN approach to estimate the soil erodibility and suggested the high performance of predictive models with R^2^ values of 0.792 and 0.815, respectively.

**Fig. 12:**
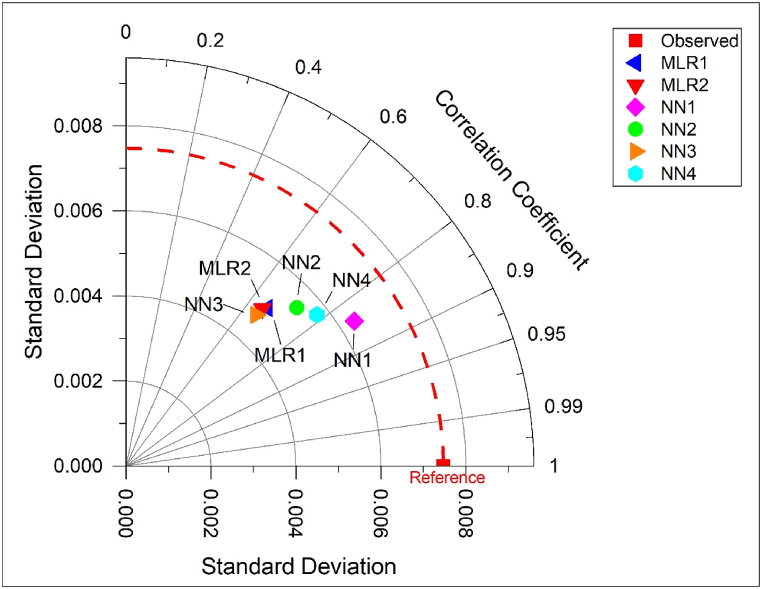
Taylor diagram for the developed models.

The efficiency of developed models was determined by R^2^, RMSE, ME, and NSE and the summary is presented in Table 5 and Table 6 for MLR and ANN models, respectively. The pattern of R^2^ values between the estimated and calculated values was in the order of NN-3>NN-1>NN-2>NN-4>MLR-1>MLR-2. RMSE was lower for all ANNs than MLRs and NSE was higher for ANNs than MLRs except NN-3. The pattern of NSE between the estimated and calculated values was in the order of NN-1>NN-4>NN-2>MLR-1>MLR-2>NN-3. The comparison of developed models using the Taylor diagram is illustrated in Fig. 12. The Taylor diagram offers a graphical representation to summarize how a developed model pattern resembles the observation [71] based on correlation coefficient and standard deviation. The diagram depicts the standard deviation as the radial distance from the point of origin whereas the azimuthal axis represents the correlation coefficient. The plotted points for developed models in closer proximity to the reference line along the arc towards the higher correlation coefficient value demonstrate optimal model performance [72,73]. From Fig. 12 it can be observed that the model NN-1 plots closest to the reference line along the higher correlation coefficient value, indicating model NN-1 to be the most optimal predictive model.

**Table 5:** Summary of efficiency indices for MLR estimation models.

Model	R	R^2^	R^2^_adj_	MSE	RMSE	NSE
MLR-1	0.668	0.446	0.423	0.0000306	0.0059	0.446
MLR-2	0.656	0.430	0.400	0.0000315	0.0058	0.430

**Table 6:** Summary of efficiency indices for ANN estimation models.

Model	R^2^	MSE	RMSE	NSE
NN-1	0.894	0.0000158	0.004	0.713
NN-2	0.855	0.0000261	0.0051	0.527
NN-3	0.940	0.0000318	0.0056	0.424
NN-4	0.826	0.0000216	0.0047	0.609

Referring to the error indices, the lower values of MSE and RMSE, while a higher value of NSE indicate better performance of a model. The high R, R^2^, and NSE values, while lesser MSE and RMSE values depict good performance and higher accuracy of a predictive model [74,75]. Even though a high R^2^ value is often preferred, however, discrepancies in the performance of predictive models may occur due to solely relying on the R^2^ value. Therefore, it is crucial to consider other metrics such as MSE, RMSE, and NSE to have a clearer understanding of the model performance. In this study, the model NN-3, despite the high R^2^ value, depicted higher MSE, RMSE, and lesser NSE values as compared with other models. Also, the model NN-3 plotted farthest from the reference line along the lower correlation coefficient, indicating the low performance of the model. This refers to the fact that despite having a higher coefficient of determination value, which shows a strong linear relationship between the observed and predicted values, the model NN-3 does not provide efficient information on the magnitude of errors. The higher values of MSE and RMSE suggest that the predicted values deviate more from the observed values and the lower NSE suggests poor performance of the model in terms of capturing the variability in the observed data.

The overall trend of efficiency indices suggests that ANN outperformed the MLR and turned out to be more efficient in forecasting of K factor in this study. For the current study, the NN-1 model, having the highest factor loading influencing factors from PCA as input variables and trained with the Levenberg-Marquardt algorithm, demonstrated lower modelling errors and better accuracy. The study targeted the development of reliable models based on artificial intelligence to efficiently predict soil erodibility based on available soil parameters. The study also provides insight about the performance and influence of soil parameters being used to develop the estimation models. The produced results from the research could be useful in the selection of appropriate soil parameters and estimation models for further predictive studies and soil erosion mitigation planning.

## 4 Conclusions

This study aims to evaluate the effectiveness of predictive models in estimating soil erodibility based on available soil parameters and indices for the selected sites in Peninsular Malaysia. The variability of the K factor and its influencing parameters was analyzed. Based on the PCA and correlation matrices, the four parameters including ***τ***_c_, FS, CLOM, and EI_ROM_ were observed to be the most influential predictors in forecasting the K factor in this study. A comprehensive assessment of these predictor variables was carried out by developing four feedforward back-propagated artificial neural networks (NN-1, NN-2, NN-3, and NN-4) and two MLR (MLR-1 and MLR-2) models for forecasting K factor. The model accuracy and efficiency indices demonstrated the better performance of ANN models in erodibility prediction, which outperformed MLR models. For this study, The NN-1 model, trained with the LM training algorithm, had higher accuracy (R^2^ = 0.894, NSE = 0.713) and lower modelling errors (MSE = 0.0000158, RMSE = 0.004) compared with MLR models and other ANN models, and hence proved out to be the best performing model for soil erodibility estimation in this study. Although the developed models demonstrated the potential for erodibility estimation, these are valid only within the considered ranges of input parameters, and beyond these ranges the models must be verified. Since in this study, the majority of influencing parameters and indices were related to soil particle size and texture, future studies may include environmental, geographical, and topographical variables. Additionally, the integration of advanced machine learning techniques and hybrid models would further refine the predictive accuracy. Moreover, the comprehensive validation of models across diverse topographic conditions and soil types would improve the applicability of predictive models. In essence, this research study offers the fundamental information and methodological support for the K factor estimation in Peninsular Malaysia.

## Funding

This work was supported by the Fundamental Research Grant Scheme (FRGS) from the 10.13039/501100002385Ministry of Higher Education (MOHE), Malaysia [FRGS/1/2021/TK0/UKM/02/11].

## Data availability statement

Data will be made available on request.

## CRediT authorship contribution statement

**Muhammad Ali Rehman:** Writing – review & editing, Writing – original draft, Software, Methodology, Investigation, Formal analysis. **Norinah Abd Rahman:** Writing – review & editing, Supervision, Resources, Funding acquisition, Conceptualization. **Ahmad Nazrul Hakimi Bin Ibrahim:** Writing – review & editing, Software, Formal analysis. **Norashikin Ahmad Kamal:** Writing – review & editing, Resources, Methodology. **Asmadi Ahmad:** Writing – review & editing, Resources.

## Declaration of competing interest

The authors declare that they have no known competing financial interests or personal relationships that could have appeared to influence the work reported in this paper.
